# β-Thalassemia gene editing therapy: Advancements and difficulties

**DOI:** 10.1097/MD.0000000000038036

**Published:** 2024-05-03

**Authors:** Jing Hu, Yebing Zhong, Pengxiang Xu, Liuyan Xin, Xiaodan Zhu, Xinghui Jiang, Weifang Gao, Bin Yang, Yijian Chen

**Affiliations:** a The First Clinical College, Gannan Medical University, Ganzhou, Jiangxi, China; b Hematology Department, The First Affiliated Hospital of Gannan Medical University, Ganzhou, Jiangxi, China.

**Keywords:** β-globin, β-thalassemia, CRISPR/Cas9, gene therapy, HBB, TALENs, traditional treatment

## Abstract

β-Thalassemia is the world’s number 1 single-gene genetic disorder and is characterized by suppressed or impaired production of β-pearl protein chains. This results in intramedullary destruction and premature lysis of red blood cells in peripheral blood. Among them, patients with transfusion-dependent β-thalassemia face the problem of long-term transfusion and iron chelation therapy, which leads to clinical complications and great economic stress. As gene editing technology improves, we are seeing the dawn of a cure for the disease, with its reduction of ineffective erythropoiesis and effective prolongation of survival in critically ill patients. Here, we provide an overview of β-thalassemia distribution and pathophysiology. In addition, we focus on gene therapy and gene editing advances. Nucleic acid endonuclease tools currently available for gene editing fall into 3 categories: zinc finger nucleases, transcription activator-like effector nucleases, and regularly interspaced short palindromic repeats (CRISPR-Cas9) nucleases. This paper reviews the exploratory applications and exploration of emerging therapeutic tools based on 3 classes of nucleic acid endonucleases in the treatment of β-thalassemia diseases.

## 1. Introduction

β-Thalassemia is an inherited disorder of hemoglobin (Hb) production, resulting in ineffective erythropoiesis.^[1]^ Chronic hemolysis is the predominant symptom, with clinical signs of myeloproliferative, extramedullary hematopoiesis, and iron homeostasis disorders.^[1]^ Patients with thalassemia suffer from the most common monogenic hereditary disease worldwide and also represent the largest monogenic hereditary disease in China.^[2]^ It originates from the Mediterranean, the Middle East, and Southeast Asia. The disease has a widespread distribution among populations and affects a large number of individuals, with an estimated carrier rate of the β-thalassemia gene at around 1.5% globally.^[3]^ β-Thalassemia is classified as nontransfusion dependent β-thalassemia or transfusion-dependent β-thalassemia (TDT) on the basis of transfusion requirements.^[4]^ Regular transfusion is the mainstay of treatment for patients with TDT; however, it brings with it iron loading related to transfusion, increased irreversible end-organ toxicity, and adverse effects of iron chelators. Hematopoietic stem cell transplantation is the only treatment, and gene editing therapy has opened the door to a cure for β-thalassemia patients. In recent years, gene editing has been developed rapidly and applied widely, such as applying mature gene-addition technology, directly repairing defective β-pearl protein genes on autologous HSCs such as zinc finger nucleases (ZFNs), transcription activator-like effector nucleases (TALENs), and especially the emerging CRISPR-Cas9 genome editing technology, or activating other genes that can replace the function of β-pearl proteins. Here, we review the emerging gene therapy modalities for the treatment of thalassemia and the possibilities for future directions.

## 2. Pathology and classification

β-Thalassemia is an autosomal recessive disorder in which the balance between β and α beads is disrupted mainly due to a reduction or deletion of the β bead protein chain. Adult hemoglobin (α2β2) is composed of either globin or β-globin. In β-thalassemia, an increase in free α-globin due to a decrease or absence of β-globin produces reactive oxidants and cellular precipitation.^[5]^ β-Thalassemia patients, due to the reduction or absence of β-globin, experience an accumulation of free alpha-globin, resulting in the generation of reactive oxygen species and cellular precipitation.^[1]^ The disease severity is categorized into 3 types: mild thalassemia, intermediate thalassemia, and severe β-thalassemia. Mild and intermediate thalassemia present with milder degrees of anemia. In thalassemia major, the unpaired bead protein chain is unstable, which results in a shorter red blood cell cycle, hemolytic precipitation, vascular occlusion, irreversible damage to organs, and a shorter survival period. The patients are also classified into transfusion-dependent thalassemia and nontransfusion-dependent thalassemia based on the assessment of their need for blood transfusion.^[5]^

Gene therapy has opened new prospects for treating β-thalassemia, aiming to restore red blood cell function and ameliorate anemia by repairing mutated genes. The directions for gene therapy in β-thalassemia primarily involve 3 approaches: first, using either the patient’s own or a donor’s healthy HSCs through a carrier-mediated method for treatment; second, employing revolutionary methods like CRISPR-Cas9 and other genome editing technologies to directly repair the defective β-globin gene in the patient’s own HSCs; and third, activating other genes capable of substituting for β-globin function, such as γ-globin or artificially synthesized β-globin. With the advancements in gene editing technologies, utilizing hematopoietic stem cells and progenitor cells (HSPCs) possessing prolonged self-renewal and self-differentiation capabilities has emerged as a novel therapeutic approach for treating β-thalassemia patients.^[6]^

## 3. Gene editing technology

**Figure 1.**
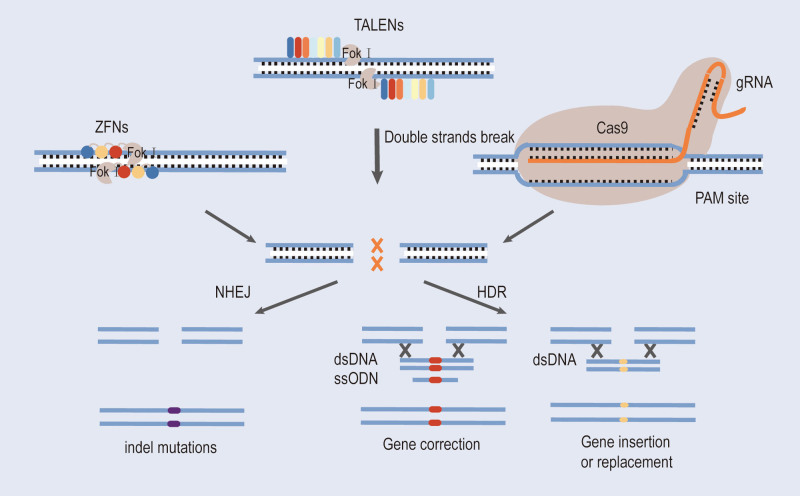
Nuclease gene editing modalities. zFNs, TALENs, and CRISPR/Cas9 mediate genome modifications through 2 major double-strand break repair pathways. Insertion deletion mutations are caused by the NHEJ pathway. FokI, nucleic acid endonuclease from *Flavobacterium*. dsDNA = double-stranded DNA, HDR = homology-directed repair, NHEJ = nonhomologous end joining, PAMs = protospacer adjacent motifs, ssODN = single-stranded oligodeoxynucleotide.

With the development of science and technology, HSPC technology is constantly being modified and refined, while nucleic acid endonuclease has the advantages of instantaneous expression, efficient delivery in vivo and ex vivo, no genome integration, a low off-target rate, high editing efficiency.^[7]^ By integrating nucleases’ technology with HSPCs, it is possible to achieve highly precise and efficient gene editing in thalassemia, ultimately accomplishing the goals of gene therapy. This article will introduce 3 types of nucleases used for gene editing: CRISPR/Cas9, TALENs, and ZFNS. It will also explore extended techniques of CRISPR technology, such as base editors and prime editor (PE). Furthermore, it will delve into the applications of these gene editing tools in thalassemia. Gene editing involves inserting the target gene through homologous recombination, but it encounters the challenge of relatively low efficiency.^[8]^ These 3 nucleases can recognize and cut specific DNA sequences in the genome, leading to double-strand breaks.^[9]^ These breaks can be efficiently repaired through either nonhomologous end joining (NHEJ) or homology-directed repair^[10]^ (Fig. 1). NHEJ is an error-prone, template-independent DNA repair mechanism that often leads to insertions or deletions of bases, potentially causing frame-shift mutations. It can alter the open reading frame, leading to the production of nonfunctional proteins and consequently resulting in functional gene knockout. Additionally, NHEJ can partially restore protein function by deleting frame-shift mutations containing exons. Unlike the error-prone NHEJ pathway, homology-directed repair-mediated gene editing achieves precise modifications by integrating donor DNA with homologous sequences into the target gene.^[11]^ This repair method is less prone to errors, facilitating precise gene therapy. Targeted gene correction in HSPCs allows for the stable, long-term expression of the corrected gene within these cells and their subpopulations. This approach ensures higher safety and endogenous expression of the target gene within the corrected HSPCs, thus enhancing the therapeutic outcome. For β-thalassemia patients, gene therapy methods are proposed mainly to correct the β-pearl protein deficiency. Gene therapy is based on gene editing techniques, which entail replacing abnormal genes with functionally expressed genes. Targeted gene correction of HSPC mutated genes resulted in long-term stable expression of the target genes in the repaired HSPC and its subcellular populations, ensuring higher safety and endogenous expression of the target genes. A proposed gene therapy approach for β-thalassemia patients focuses on correcting β-globin deficiency. Gene therapy is based on gene editing technology, which requires the replacement of abnormal genes with functionally expressed genes.

### 3.1. The application of nucleases in β-thalassemia

Gene editing technology relies on nucleases, and based on their structure, the nucleases used for gene editing can be classified into 3 types: ZFNs, TALENs, and CRISPR-associated nucleases.^[12]^

ZFNs are composed of the DNA-binding domains of transcription factor ZFPs and the nuclease domains of the FokI restriction endonuclease, and they exert their function.^[13]^ The designed zinc finger domains’ binding specificity guides ZFNs to specific genomic loci, enabling specific cleavage at particular sites within the genome. ZFNs were the first nucleases used in clinical experiments.^[12]^ In a gene therapy attempt for β-thalassemia using ZFNs technology, the researchers introduced a mutation in the SOX6 binding region, which exerted its effect through lentiviral vectors to reactivate γ-bead protein gene expression. Five days after red lineage differentiation, γ-bead protein mRNA levels were 6 times higher in ZFN-treated cells than in untreated cells, while fetal hemoglobin (HbF), measured using hemoglobin electrophoresis, was found to be elevated in expression levels as well. Additionally, using hemoglobin electrophoresis to measure HbF, they found an elevated expression level.^[14]^ ST-400 consists of autologous CD34 + cells, which, after precise ex vivo editing mediated by high-precision ZFNs on the erythroid-specific enhancer of BCL11A, enable efficient expression of HbF.^[14]^ The experiment recruited 6 patients and employed autologous enrichment of CD34 + cells, transfecting them with mRNA encoding ZFNs to enhance HbF expression in the erythroid lineage during TDT. The binding sites of ZFNs were located on both sides of the GATA-binding region within BCL11A erythroid-specific enhancer. Two patients who underwent myeloablative conditioning experienced rapid hematopoietic reconstitution post-HSCGT, showing increased levels of HbF after the hematopoietic stem cell gene therapy.^[14]^ ZFNs were the earliest nucleases used for precise gene editing, known for their simplicity in design. However, their major drawback lies in their editing specificity, which relies not only on the target sequence itself but also on adjacent sequences within the genome. This reliance may cause genomic instability and potential breaks, limiting their wide applicability.^[15]^

TALENs operate with the same simplicity and flexibility as ZFNs. They also contain DNA-binding and cleavage domains, allowing precise targeting of specific regions within the genome.^[16]^ Due to the easier design of the DNA-binding domains in TALEs compared to zinc finger proteins, TALENs have a broader application in life sciences than ZFNs.^[17]^ In 2013, TALENs were used to correct β-globin gene mutations in induced pluripotent stem cells derived from 2 β-thalassemia patients. Researchers induced differentiation of induced pluripotent stem cells lines from each patient into hematopoietic progenitor cells, subsequently differentiating them into red blood cells expressing normal β-globin. The study demonstrated that the targeted gene correction process did not produce any TALEN-induced off-target mutations.^[18]^ TALENs technology was also used to correct the IVS2-645C > T mutation in the β-globin gene. Compared to the uncorrected control, red blood cells derived from corrected iPSCs using TALENs showed increased expression of the β-globin gene.^[19]^ TALENs vectors targeting the human β-globin gene mutation IVS2-645C > T were introduced into mice, resulting in the generation of biallelic TALENs+/β^654^ mice. DNA sequencing analysis revealed deletion of this point mutation in over 50% of TALENs+/β^654^ mice. Further RT-PCR and Western blot analysis confirmed normal expression of the β-globin gene in these mice. They exhibited significantly reduced splenomegaly, and reduced iron deposition in the liver and spleen.^[20]^

CRISPR-Cas9 nucleases are part of a bacterial immune system capable of cleaving phage or plasmid DNA. Their function is akin to “scissors,” enabling precise cutting at specific DNA sites within the genome.^[21]^ The CRISPR-Cas system recognizes protospacer adjacent motifs and target-specific CRISPR RNA (crRNA). CRISPR-Cas nucleases use RNA to guide them to specific DNA sequences, allowing them to form base pairs and cut foreign genetic segments, thereby protecting the host from infections.^[21]^ BCL11A is a zinc finger-containing transcription factor that suppresses the expression of γ-globulin and HbF in red blood cells.^[22]^ Consequently, reducing the expression of BCL11A can increase the expression of HbF, restoring the balance between alpha-globin and β-globin and ultimately restoring red blood cell function. Scientists have successfully employed CRISPR-Cas9 gene editing technology in hematopoietic stem and progenitor cells (HSPCs), reducing the expression of BCL11A in the erythroid lineage. This led to the restoration of γ-globulin synthesis, reactivating the production of HbF.^[23]^ Cas9, a widely used gene editing tool, has corrected the HBB IVS2-654 mutation in induced pluripotent stem cells, restoring normal expression of the HBB gene. Compared to TALENs, Cas9 results in a more stable and straightforward genome after correction, which is among its broad advantages in recent years. Furthermore, based on CRISPR-Cas9 gene editing technology, gene editing drugs have successfully reached the market. Dr Frangoul utilized CRISPR technology to edit CD34 + HSPCs, producing CTX001. Its ability to reactivate HbF production positions it as a potential clinical drug for treating β-thalassemia. The experimental outcomes of targeted therapy are promising.^[24]^ A high frequency of allelic editing was observed with the CTX001 product in CD34 + hematopoietic stem cells (HSPCs) from 10 healthy donors, with an average of 80% (±6%) covering all subpopulations of CD34 + cells and an average fetal hemoglobin level of 29.0% (±10.8%) in the edited cells.^[25]^ CTX001 has entered phase III clinical trials (Table 1). Another phase 1/2 trial (NCT04211480) effectively demonstrated its lasting cellular editing and safety. In 2 pediatric patients, the persistence of bone marrow cell editing was over 85%. There was a significant increase in Hb levels, and these patients didn’t require transfusion therapy for 18 months posttreatment.^[26]^

**Table 1:** Clinical trials of CRISPR.

Status	Study title	Intervention	Phases	Information
ACTIVE_NOT_RECRUITING	A Safety and Efficacy Study Evaluating CTX001 in Subjects With Transfusion-Dependent β-Thalassemia	BIOLOGICAL: CTX001	Phase 2 Phase 3	This is a single-arm, open-label, multi-site, single-dose Phase 1/2/3 study in subjects with TDT. The study will evaluate the safety and efficacy of autologous CRISPR-Cas9 Modified CD34 + Human Hematopoietic Stem and Progenitor Cells (hHSPCs) using CTX001
RECRUITING	Evaluation of Efficacy and Safety of a Single Dose of CTX001 in Participants With Transfusion-Dependent β-Thalassemia and Severe Sickle Cell Disease	BIOLOGICAL: CTX001	Phase 3	This is a single-dose, open-label study in participants with TDT or severe sickle cell disease (SCD). The study will evaluate the safety and efficacy of autologous CRISPR-Cas9 modified CD34 + hHSPCs using CTX001
ACTIVE_NOT_RECRUITING	A Safety and Efficacy Study Evaluating ET-01 in Subjects With Transfusion Dependent β-Thalassemia	BIOLOGICAL: ET-01	Phase 1	This is a single-arm, open-label, multi-center, single-dose phase 1 study in subjects with transfusion dependent β-thalassemia. The study will evaluate the safety and efficacy of autologous CRISPR-Cas9 Modified CD34 + hHSPCs using ET-01.
RECRUITING	Safety and Efficacy Evaluation of BRL-101 in Subjects With Transfusion-Dependent β-Thalassemia	DRUG: BRL-101	Phase 1	This is a nonrandomized, open-label, multi-site, single-dose, phase 1/2 study in subjects with TDT. The study will evaluate the safety and efficacy of autologous CRISPR-Cas9 modified CD34 + hHSPCs (BRL-101)
RECRUITING	Evaluation of Safety and Efficacy of CTX001 in Pediatric Participants With Transfusion-Dependent β-Thalassemia (TDT)	BIOLOGICAL: CTX001	Phase 3	This is a single-dose, open-label study in pediatric participants with TDT. The study will evaluate the safety and efficacy of autologous CRISPR-Cas9 modified CD34 + hHSPCs (CTX001)
UNKNOWN	iHSCs With the Gene Correction of HBB Intervent Subjects With β-Thalassemia Mutations	BIOLOGICAL: iHSCs treatment group	Early Phase 1	This is a single center、single-arm、open-label study，to investigate the safety and efficacy of the gene correction of HBB in patient-specific iHSCs using CRISPR/Cas9
ENROLLING_BY_INVITATION	A Long-term Follow-up Study in Subjects Who Received CTX001	BIOLOGICAL: CTX001	Phase 3	This is a multi-site, observational study to evaluate the long-term safety and efficacy of CTX001 in subjects who received CTX001 in Study CTX001-111 (NCT03655678) or VX21-CTX001-141 TDT studies or Study CTX001-121 (NCT03745287) or VX21-CTX001-151 SCD studies; NCT05329649)

The spreadsheet contains all the basic information related to CRISPR-Cas9 gene therapy for β-thalassemia listed on the official website (https://β.clinicaltrials.gov/). It includes publication titles and clinical stages.

The main purpose of gene editing is to reduce the production of ineffective red blood cells, decrease extramedullary hematopoiesis, enhance editing efficiency, and mitigate adverse consequences arising from base insertions. Of course, there are shortcomings, too. If mutant cells are present and persist in a population, the result is a deviation from the target, like a snowball effect, and it increases over generations.^[27]^ In 2023, experts proposed combining CRISPR with nanoparticle signal transduction as a means to detect nucleic acid sequences, potentially avoiding off-target effects.^[28]^ There are several methods to reduce off-target gene editing. Firstly, 1 approach involves optimizing the paired cleavage sites to enhance the specificity of gene editing.^[26]^ Secondly, enhancing off-target detection efficiency and optimizing detection tools. For instance, the discovery that tagging Cas9 with a ubiquitin-proteasome degradation signal can facilitate Cas9 degradation in nonhuman primate embryos, reducing unintended genomic insertions.^[29]^ Also, in genetic editing research, zebrafish serve as an ideal model for blood-related studies. Due to their short growth cycle, high reproductive numbers, and a molecular mechanism of red blood cell generation highly similar to that of mammals, they can be used as a method to screen for errors that occur during genetic editing.^[30]^ CRISPR considers single nucleotide polymorphisms and insertions and deletions in genetic variations. Validated with SpCas9 in CD34 HSPCs, this method exhibits high feasibility and holds promise as a tool for validating gene editing efforts in the future.^[31]^

### 3.2. Exploration of CRISPR/Cas9 new technology

CRISPR, as the most promising gene editing technology, is widely used due to its simpler gene design and lower costs compared to the aforementioned nucleases. In recent research advancements, Dr Trakarnsanga’s team pioneered the creation of immortalized red blood cell lines (SiBBE) using the CRISPR/Cas9 system to correct the HbE gene and restore β-globin production. This innovative approach addresses the challenge of sustaining efficiently corrected red blood cells, offering another pathway for future gene therapy in treating β-thalassemia.^[32]^ Another technological derivative, the CRISPR-derived PE, represents a functional extension of the CRISPR system. PE utilizes these tools for promoter editing and epigenetic regulation, among other directions.^[27]^ In mouse experiments, PE was used to correct dysfunctional β-globin genes, achieving a gene editing efficiency of 14.29%. Following correction, the mice exhibited no abnormalities in red blood cells and no extramedullary hematopoiesis.^[33]^ Craspase is a dual-gene editor within the CRISPR/Cas system. Its key feature lies in enabling precise editing of RNA or protein sequences while significantly reducing off-target effects and cellular toxicity.^[34,35]^ This technology holds the potential to substantially improve current gene editing capabilities and provides a possibility for novel targeted RNA editing. The CRISPR technology isn’t solely for correcting genes but can also be used to evaluate gene editing efficiency and variations. CRISPRme serves as a robust tool for assessing variations and off-target effects. It considers single nucleotide polymorphisms and insertions/deletions in genetic variations. Dr Pinello validated this in HSPCs and discovered its potential to alleviate off-target effects. It effectively demonstrates how modifying factors impact genetic variations.^[31]^

### 3.3. Gene addition

In the gene-addition therapy process, 2 key players are the lentiviral vector and the functional β-globin gene. Gene addition is the fusion of a lentivirus with a host cell, followed by the transcription of the lentivirus’ RNA genome into DNA by reverse transcriptase and the integration of this DNA into the host genome. Its shortcoming is the insertion time of lentiviral vectors. However, the LentiGlobin BB305 gene add-on treatment enabled patients to produce stable functional adult hemoglobin and become transfusion independent. In a clinical study involving 22 transfusion-dependent β-thalassemia patients who underwent ex vivo transduction of cells with the LentiGlobin BB305 vector, it was observed that around 26 months posttreatment, severe thalassemia patients ceased transfusions, and there were increases observed in HbA ^T87Q^ levels and total hemoglobin levels.^[36]^ In a 6-year follow-up study, patients involved in Phase 3 treatment had a longer transfusion-free period compared to those in Phase 1/2. They didn’t experience any treatment-related adverse events, lentivirus replication, or secondary tumors.^[36]^

## 4. Other treatments for β-thalassemia

Human leukocyte antigen-matched allogeneic hematopoietic stem cell transplantation has been continuously maturing and showing promise in treating β-thalassemia patients. The key challenge with allogeneic stem cell transplantation lies in donor limitations, leading to acute and chronic graft vs host disease and high costs. Haploidentical stem cell transplantation aims to overcome human leukocyte antigen limitations by broadening the donor pool, but its efficacy remains limited. Umbilical cord blood stem cells represent another alternative to allogeneic stem cells, but they often have lower hematopoietic stem cell concentrations, leading to transplant failure.^[37]^ Reactivating HbF is a promising approach for symptom improvement. Hydroxyurea (HU) is a classic inducer of HbF.^[38]^ Before and after its use, there have been increases observed in hemoglobin (Hb), average Hb, and HbF levels.^[39]^ Hydroxyurea generally shows good therapeutic efficacy, but there are instances of partial drug resistance. Luspatercept, a recombinant fusion protein, aids in promoting red blood cell maturation and alleviating the need for transfusions in β-thalassemia. In mouse erythroleukemia cells in vitro, ligand-mediated Smad2/3 pathway reduction of GATA-binding factor-1 and its transcriptional activator TIF1γ decreases red cell differentiation, mitigating ineffective erythropoiesis.^[40]^ In a Phase 3 clinical trial, the overall effectiveness of Luspatercept was higher compared to the control group, although some adverse events were reported.^[41]^ Roxadustat is a potential treatment for β-thalassemia. It functions by targeting hypoxia-inducible factor, primarily regulating pathways involving transforming growth factor-β and erythropoietin (EPO) to stimulate red blood cell production.^[42]^ The 3 medications mentioned can increase HbF levels and reduce transfusion requirements in severe β-thalassemia patients. However, they cannot address the root cause of the disease, leading to ongoing challenges related to long-term transfusions and iron overload.

## 5. Questions and prospects

Gene therapy for β-thalassemia has evolved from single hematopoietic stem cell repair to next-generation gene therapy techniques, providing more accurate treatment not only for thalassemia patients but also for individuals with single-gene disorders. This involves using carrier-delivered normal HSCs from either the patient or a donor, or employing genome editing technologies like ZFNs, TALENs, CRISPR-Cas9, etc, to directly repair the defective β-globin gene in autologous HSCs or activate other genes capable of substituting for β-globin function. Despite the challenges gene editing such as low delivery efficiency, editing rates, and potential off-target mutations, its advantages remain particularly significant. Combining gene editing techniques with zebrafish models and single-cell sequencing technologies can mitigate off-target gene editing. With advancements in gene editing research, gene therapy is expected to offer greater advantages in treating β-thalassemia patients. Moreover, there is widespread support for repairing individual human cell DNA.^[43]^ At present, the risks associated with germline gene editing are not fully understood, therefore germline gene editing cannot be pursued.^[44]^ This review primarily focuses on 3 types of gene editing based on nucleases and their application in β-thalassemia. It details advancements in genome editing technologies, emphasizing precision and efficiency as the preferred methods for treating β-thalassemia. The review underscores the significant prospects for future developments in this field.

Compared to conventional treatments, utilizing gene therapy for β-thalassemia can reduce the risk of iron overload and its complications from long-term blood transfusions, as well as minimize ineffective red blood cell production. Notably, CRISPR/Cas9-mediated gene editing for β-thalassemia has shown promising evidence. Trials such as the ongoing Phase 1/2 trial in China (NCT04211480) and outcomes from the RM-001 gene product are exciting, offering hope for potential cures for suitable patients in the future.^[26,45]^

## Author contributions

**Data curation:** Liuyan Xin, Xiaodan Zhu, Xinghui Jiang.

**Investigation:** Weifang Gao, Bin Yang.

**Software:** Pengxiang Xu.

**Validation:** Yijian Chen.

**Visualization:** Yebing Zhong.

**Writing – original draft:** Jing Hu.

**Writing – review & editing:** Jing Hu, Yijian Chen.
